# INTERGENERATIONAL LEARNING TO ADDRESSING AGEISM IN HIGHER EDUCATION: SCOPING REVIEW FINDINGS

**DOI:** 10.1093/geroni/igad104.1094

**Published:** 2023-12-21

**Authors:** Michelle Hand, Catherine Tompkins, Emily Ihara, Megumi Inoue, Hyun Kang, Kaitlyn Harvey

**Affiliations:** George Mason University, Fairfax, Virginia, United States; George Mason University, Fairfax, Virginia, United States; George Mason University, Fairfax, Virginia, United States; George Mason University, Fairfax, Virginia, United States; George Mason University, Fairfax, Virginia, United States; George Mason University, Fairfax, Virginia, United States

## Abstract

Intergenerational learning strategies can increase wellness, both among students and older community members, while challenging ageism on college and university campuses. Still, existing studies on the mutual benefits and effectiveness of intergenerational learning strategies for addressing biases among college and university students are limited and as such, are continuing to emerge. Thus, a scoping review was conducted to explore current research on intergenerational learning in higher education, the potential relevance of such strategies to reduce ageism and better prepare future service providers, and to explore current implications for education, practice, policy, and research. Included in this scoping review were peer-reviewed and grey sources based on primary research on what is known about intergenerational learning strategies in higher education and their potential impacts on ageism. Excluded were sources not based on primary research or without clear implications for relevant education, practice, policy or research. In total, 313 articles were identified; 67 of these were screened in for full text reading, and 37 were initially screened in for analysis, after which another 8 were excluded, resulting in 29 cases for thematic analysis. Key themes surround intergenerational service learning and creative expression, attitudes toward older adults, perceptions on aging, mutual benefits of intergenerational learning (e.g., cultural learning and integration), and remaining needs for research and education, through integrating service learning opportunities into existing programs and courses in higher education. The results of this scoping review will be further discussed, along with future implications for research, practice, policy and education focused on reducing ageism.

